# Effects of Genetic Variation on Urinary Small Molecule Signatures of Mice after Exposure to Ionizing Radiation: A Study of p53 Deficiency

**DOI:** 10.3390/metabo10060234

**Published:** 2020-06-08

**Authors:** Evan L. Pannkuk, Evagelia C. Laiakis, Pelagie Ake, Steven J. Strawn, Yi-Wen Wang, Albert J. Fornace

**Affiliations:** 1 Department of Oncology, Lombardi Comprehensive Cancer Center, Georgetown University Medical Center, Washington, DC 20057, USA; elp44@georgetown.edu (E.L.P.); ecl28@georgetown.edu (E.C.L.); pya@georgetown.edu (P.A.); 2 Department of Biochemistry and Molecular & Cellular Biology, Georgetown University Medical Center, Washington, DC 20057, USA; steve.strawn@gmail.com (S.J.S.); yiswe421@gmail.com (Y.-W.W.)

**Keywords:** biodosimetry, p53, ionizing radiation, urine, metabolomics, acute radiation syndrome, mass spectrometry

## Abstract

Due to risks from potential exposures to ionizing radiation (IR), improved radiological countermeasures are required, as well as rapid high-throughput biodosimetry. Genotypic variation in the general population contributes to differences in radiosensitivity that may affect biodosimetry accuracy. Previous studies utilized radiosensitive mutant mouse models (Parp1^−/−^ and Atm^−/−^) to determine the effects of genotypic deficiency on radiation signatures. Here, we extend this approach by examining changes in the urinary metabolome in a hematopoietic (HP) resistant mouse model (p53^−/−^) after IR exposure. As p53 is a primary regulator in radiation response and apoptosis, limited hematopoietic stem cell apoptosis leads to reduced mortality at doses of ~8–10 Gy but increased mortality at higher doses (>15 Gy) due to mitotic catastrophe in gastrointestinal (GI) crypt cells. Urine was collected from mice (wild-type (WT), p53^+/−^, and p53^−/−^) pre-irradiation and at 4 and 24 h after total body irradiation (TBI) (WT: 8 and 10 Gy; p53^−/−^: 10 Gy) for metabolic phenotyping using an ultra-performance liquid chromatography mass spectrometry (UPLC-MS) platform. Minimal differences were detected between unirradiated WT, p53^+/−^, and p53^−/−^ mice. While similar perturbations were observed for metabolites involved in tryptophan, vitamin B6, and histamine pathways, glycine conjugation, and redox metabolism for WT and p53^−/−^ mice after TBI, an overall dampened response was observed in p53-deficient mice. Despite comparable metabolite patterns between genotypes, differentiation was achieved through receiver operating characteristic curve analysis with high specificity and sensitivity for carnitine, N1-acetylspermidine, and creatine. These studies highlight that both attenuated and dampened metabolic responses due to genetic variability in the general population need to be addressed in biodosimetry frameworks.

## 1. Introduction

The need for rapid biodosimetry, radiological countermeasures, and mitigation efforts has arisen from the ongoing risks of potential exposures to ionizing radiation (IR), including through accidental (nuclear power plants or stored waste) or malicious (radiological terrorism) events. Concerning biodosimetry, practical biomarkers are needed that can classify the clinical severity of acute radiation syndrome (ARS), or the absence thereof, to administer appropriate medical care. While lower exposure levels (<2 Gy) would require minor medical treatment in the absence of secondary conditions, higher exposure levels may lead to hematopoietic syndrome (2–4 Gy) that may be treated with supportive care such as cytokine therapy [1]. Potential exposure may affect many thousands of individuals, requiring methods for high-throughput biodosimetry (e.g., seconds or minutes per assay) as current techniques can take hours to typically days to achieve results [2]. Those affected include individuals of varying phenotypes, including those with radiosensitivity, which will need to be factored into biomarker panel development and interpretation. In addition to radiation accidents or mass exposure events, biomarkers of radiosensitive individuals may be useful for screening prior to medical procedures, particularly radiotherapy [3].

To address the need for rapid biodosimetry, past studies have assessed the utility of metabolic phenotyping to estimate radiation injury [1]. The basis for this approach is that as IR exposure damages cells through direct interactions with biomolecules and indirectly through free radical production, a discernable small molecule signature will be produced that is predictive of ARS level. Metabolomic platforms can quickly provide relative concentration values on hundreds (e.g., proton nuclear magnetic resonance spectroscopy (^1^H-NMR) or gas chromatography (GC)-mass spectrometry (MS)) to thousands (e.g., liquid chromatography (LC)-MS) of compounds or spectral features in a given sample [4,5]. Ideally, easily accessible biofluids (urine, saliva, blood) can be used due to the logistics of sample collection and transportation in an emergency situation. While untargeted profiling with subsequent multivariate data analysis has determined discernable small molecule signatures in several animal models and humans, these signatures are continually refined to provide targeted strategies to determine the specificity and sensitivity of metabolite panels [6,7] and reduce cost and time to result [8].

Genetically engineered mouse models have proven a valuable resource for assessing how deficient DNA repair may affect radiosensitivity and biomarker panel development. Poly (ADP-ribose) polymerase 1 (Parp1) plays an integral role in DNA damage response, including single and double-strand break repair, DNA replication fork stabilization, and modulation of chromatin structure [9]. The urinary profiles of Parp1^−/−^ mice show an increased perturbation to tricarboxylic acid (TCA) cycle intermediate levels and general lower fold changes in other compounds after exposure to a semi-lethal dose of radiation (6 Gy) [10]. A rare autosomal recessive disorder, ataxia telangiectasia (AT), causes higher radiation sensitivity due to lowered DNA repair, DNA recombination, and cell-cycle control [11]. The A-T mutated (ATM) gene acts upstream to phosphorylate p53 at S^15^ and S^20^ to enable stabilization, and Atm^−/−^ mice show discernable urinary profiles after IR exposure as compared to wild-type (WT) mice [12]. The p53 protein is well known for its roles in tumor suppression, genomic stability, apoptosis, and is a primary regulator in radiation response [13,14]. A major cause of ARS lethality at higher IR doses (≥8 Gy in mice) is p53-dependent apoptosis of hematopoietic stem cells [15]. Small molecule inhibition of p53 leads to hematopoietic radioresistance, which has also been observed in p53-deficient mice [16]. Conversely, at higher IR exposures (≥15 Gy in mice) eliciting more severe gastrointestinal (GI) syndrome, lack of cell arrest leads to mitotic catastrophe in crypt cells and increased radiosensitivity [17]. p53 mutations in the general population span the most common form of alteration in human tumors to rather rare germline mutations (Li-Fraumeni syndrome (LFS)) [18]. Therefore, the inclusion of resistant mutant mouse models is needed to explore how varying radiosensitivity in the general population will affect radiation signatures and accuracy of biodosimetry models.

In this study, we analyzed urinary small molecule profiles from WT and p53^−/−^ mice after a total body irradiation (TBI) dose (WT: 8 and 10 Gy; p53^−/−^: 10 Gy) to elicit hematopoietic syndrome but not cause severe GI injury. As hypothesized, we found greater perturbation in the urinary profiles of WT mice compared to p53^−/−^ mice at 4 and 24 h. Nine validated metabolites exhibited similar trends between genotypes but were not statistically significant in p53^−/−^ mice (except N1-acetylspermidine at 24 h). One consistent metabolite indicative of IR exposure, carnitine, showed no change in p53^−/−^ mice and when combined with N1-acetylspermidine and creatine could differentiate between genotypes post-exposure with high sensitivity and specificity. These results suggest compensatory mechanisms may present genotype independent urinary biomarkers, but dampened and attenuated responses must be considered in predictive models of radiological injury.

## 2. Material and Methods

### 2.1. Animal Models and Radiation Exposure

WT C57Bl/6 and p53^−/−^ mice were obtained from The Jackson Laboratory (www.jax.org/strain/002101, [19]) and bred/irradiated at Georgetown University according to Georgetown University Institutional Animal Care and Use Committee (GUACUC) protocols (2016-1152). Mice were provided water and food ad libitum (12 h light/12 h dark cycle conditions). Mice were acclimated to metabolic cages for 24 h, and then pre-irradiation urine samples were collected over a 24-h period (Figure S1). Pre-irradiation samples were also collected from p53^+/−^ mice to further resolve genotypic differences. Male mice that were 10–12 weeks old were exposed to a TBI X-ray dose (~1.67 Gy/min; X-Rad 320, Precision X-Ray Inc, Branford, CT, USA; filter, 0.75 mm tin/0.25 mm copper/1.5 mm aluminum) of 0 Gy (sham) and 10 Gy (WT and p53^−/−^). To compare WT with lower hematopoietic apoptosis, an equitoxic dose of 8 Gy (LD_50/30_) was also included. Post-irradiation urine samples were collected in metabolic cages at 4 h or 24 h post-exposure, and immediately stored at −80 °C until further use.

### 2.2. Metabolite Extraction and LC-MS Analysis 

Reagents for sample preparation and LC mobile phases (Optima^TM^ grade) were obtained from Thermo Fisher Scientific^TM^ Inc. (Waltham, MA, USA). Authentic chemical standards were of the highest purity available: debrisoquine sulfate, 4-nitrobenzoic acid, carnitine, creatine, hippuric acid, 4-pyridoxic acid, phenylacetylglycine, 4-hydroxy-3-methoxyphenylglycol sulfate, 1-methylhistamine, 1-methylnicotinamide (Sigma-Aldrich^®^ LLC, St. Louis, MO, USA), and N1-acetylspermidine (Cayman Chemical Company, Ann Arbor, MI, USA).

Urine samples were prepared as previously described [20]. Urine (20 μL) was deproteinated with 50% acetonitrile (80 μL) containing internal standards (2 μM debrisoquine sulfate, 30 μM 4-nitrobenzoic acid), incubated on ice for 10 min, vortexed for 30 s, and centrifuged for 10 min (10,000× *g*, 4 °C). A quality control (QC) sample was prepared by mixing 1 μL of urine from each sample and prepared as above and run every 10 samples.

Samples were injected (2 μL) and analyzed by an ACQUITY UPLC (BEH C18 1.7 μM, 2.1 × 50 mm column) coupled to a Xevo^®^ G2 QTOF-MS (Waters Corp., Milford, MA, USA). Data-independent acquisition was performed in both negative and positive electrospray ionization (ESI) modes as previously described [6] using leucine enkephalin (556.2771 [M + H]^+^ or 554.2615 [M − H]^−^) as Lockspray^®^ to calibrate accurate mass.

### 2.3. Data Processing, Statistical Analysis, and Marker Validation

Data files were inspected in MassLynx v.4.1 (Waters, Milford, MA), pre-processed in Progenesis QI (Nonlinear Dynamics, Newcastle, UK), and normalized to internal standards (debrisoquine (ESI^+^, M + H = 176.1188) or 4-nitrobenzoic acid (ESI^−^, M − H = 166.0140)) as previously described [21]. The resultant data matrix was visualized with a principal component analysis (PCA) in MetaboAnalyst to assess similarities among unirradiated genotypes (WT, p53^+/−^, and p53^−/−^ mice) [22]. Positive and negative mode datasets were analyzed separately. Spectral features significantly different between WT and p53^−/−^ mice were assessed using a Welch’s *t*-test (*p* < 0.05, ≥ 70% presence in both groups, false discovery rate (FDR) corrected *p*-values determined with a classical Benjamini-–Hochberg step-up correction procedure) or a Barnard’s test (< 70% presence in a single group) using the software MetaboLyzer [23]. Heatmaps (top 100 ranked positive mode ions) were generated using the machine-learning algorithm Random Forests programmed in R v.2.15.2 [24], and volcano plots were generated using MetaboLyzer. Putative identification of the spectral features was determined by comparing monoisotopic mass (±10 ppm error) to the Human Metabolome Database (HMDB) [25], the Kyoto Encyclopedia of Genes and Genomes (KEGG) [26], and the Chemical Entities of Biological Interest (ChEBI) database [27]. Ions were validated by tandem MS (5–50 V ramping collision energy) and fragmentation patterns were compared to pure standards and compound databases (e.g., the METLIN tandem MS database) for unambiguous identification [28,29]. Validated compounds were checked for outliers and plotted in GraphPad Prism 6 (GraphPad Software, La Jolla, CA, USA), and heatmaps were generated in MetaboAnalyst. Additionally, the specificity and sensitivity of markers were determined by the area under the curve (AUC) values from receiver operating characteristic (ROC) analysis in MetaboAnalyst, with a Random Forests classification method for combined metabolites [22]. Pathway analysis and enrichment was performed in MetaboAnalyst using the KEGG, HMDB, and the Small Molecule Pathway Database.

## 3. Results

**Figure 1:**
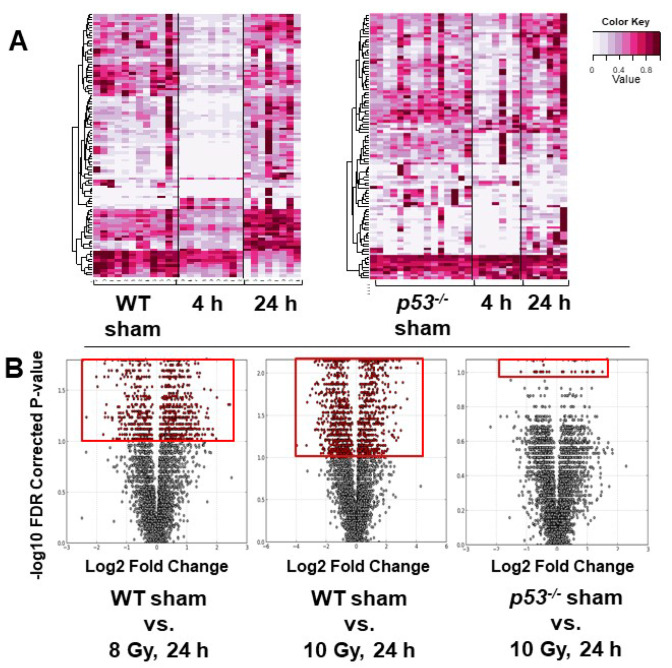
(**A**) Heatmaps generated by the machine-learning algorithm Random Forests of the top 100 ions at 4 and 24 h after 8 Gy total body irradiation for wild-type (WT) mice and at 4 and 24 h after 10 Gy for p53^−/−^ mice. More pronounced fold decreases of ions were observed at 4 h in WT compared to p53^−/−^. (**B**) Volcano plots generated from MetaboLyzer at 24 h post-irradiation for WT (8 and 10 Gy) or p53^−/−^ (10 Gy) mice. Red inserts highlight the increased number of significant spectral features, showing higher perturbation in WT after a 10-Gy ionizing radiation (IR) dose compared to 8 Gy. Fewer significant ions were present in p53-deficient mice.

Minimal cage effects were observed after a 4 or 24 h housing period for mice receiving a sham dose and were combined to serve as the control group for their respective genotype for further comparisons. Additionally, few differences between unirradiated genotypes (WT, p53^+/−^, and p53^−/−^) were observed and only irradiated WT and p53^−/−^ were further explored (Figure S2). A higher number of perturbed metabolites were identified for WT compared to p53^−/−^ mice at both 8 and 10 Gy (Figure 1). Heatmaps generated through Random Forests of the top 100 spectral features in positive ionization mode showed a general trend of lower concentrations at 4 h with increased concentrations at 24 h for both genotypes (Figure 1A), although it should be noted that the top 100 spectral features were not identical between WT and p53^−/−^ mice. Visualization with volcano plots clearly demonstrated more significant spectral features in WT vs. p53^−/−^ mice (Figure 1B).

**Figure 2:**
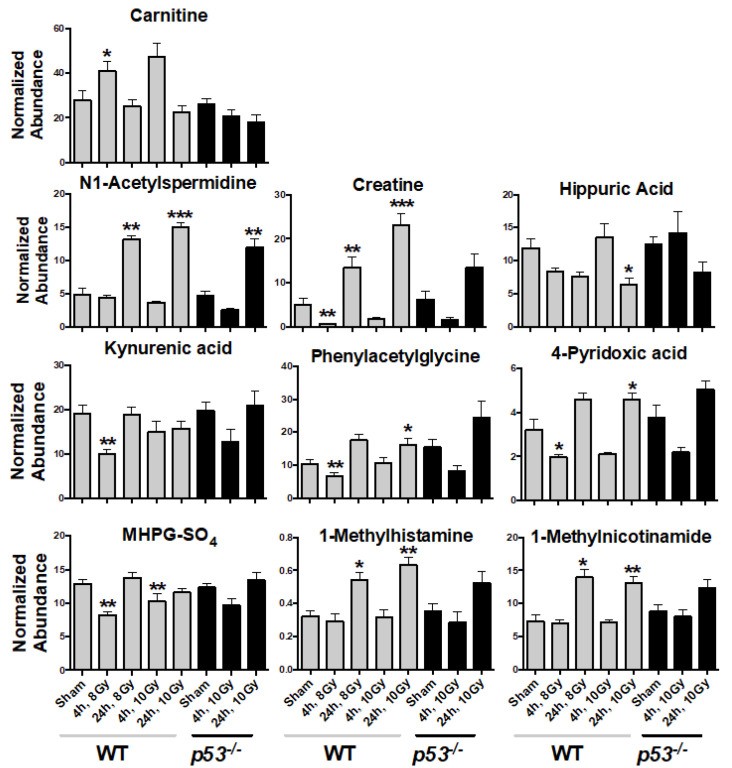
Urinary metabolite concentrations in wild-type (WT) mice and p53^−/−^ mice at 4 and 24 h post-irradiation (8 and 10 Gy WT, 10 Gy p53^−/−^). Carnitine levels were perturbed only in WT mice; however, other metabolite levels showed similar trends in p53^−/−^ mice but were not statistically significant (except N1-acetylspermidine at 24 h). (* *p* ≤ 0.05, ** *p* ≤ 0.01, *** *p* ≤ 0.001, Mean ± SE, significant spectral features were identified by Welch’s *t*-test with a false-discovery rate corrected *p* ≤ 0.05).

**Figure 3:**
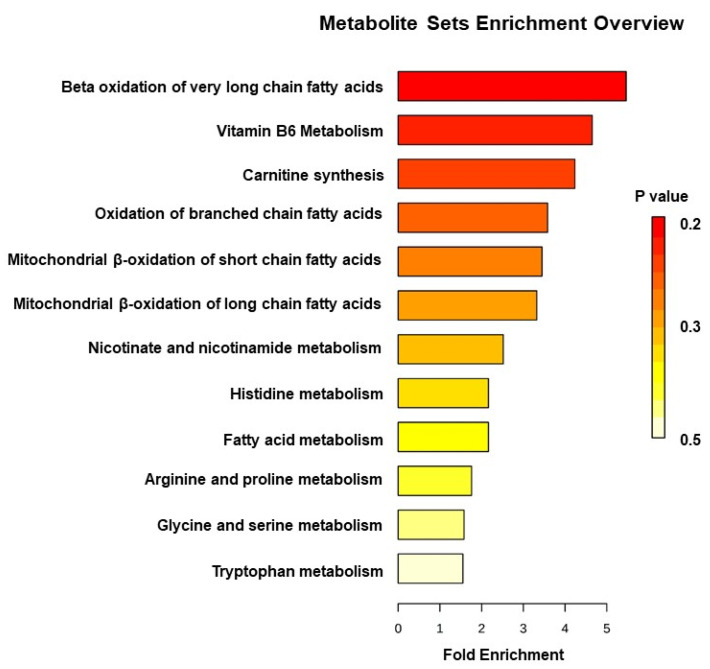
Hierarchical representation of impacted metabolic pathways identified in wild-type and p53^−/−^ mice post-irradiation. Metabolic pathways were identified in MetaboAnalyst using the Kyoto Encyclopedia of Genes and Genomes (KEGG) database, The Small Molecule Pathway Database, and Human Metabolome Database (HMDB).

**Figure 4:**
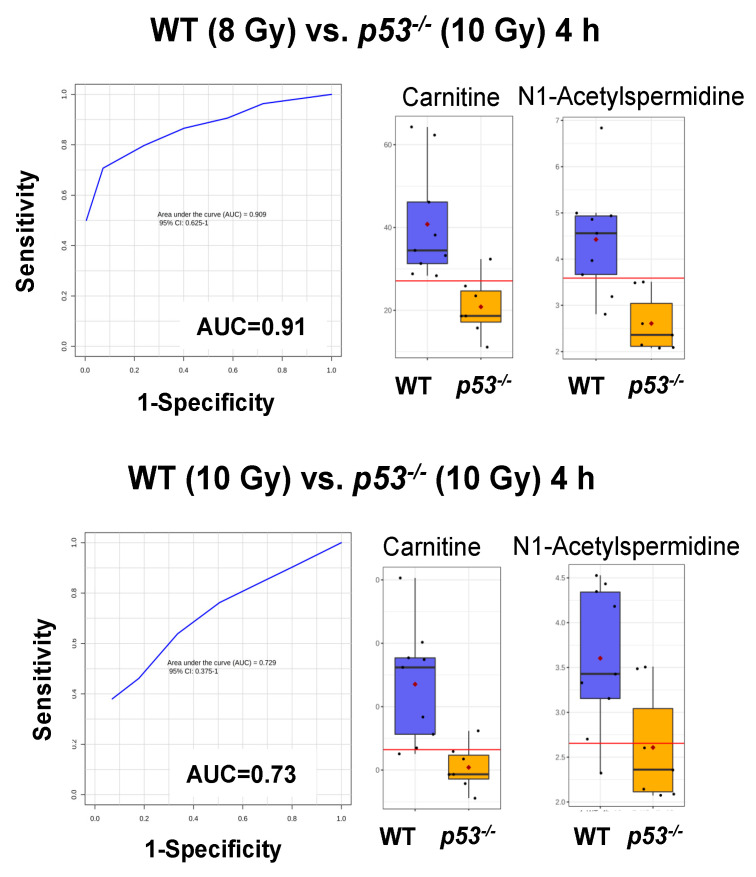
Receiver operating characteristic analysis of wild-type (WT) mice and p53^−/−^ mice. The highest specificity and sensitivity was obtained for carnitine and N1-acetylspermidine grouped at 4 h for the equitoxic dose (8 Gy) (area under the curve (AUC) = 0.91), with box and whisker plots illustrating higher concentration in WT vs. p53^−/−^ mice urine post-irradiation. Classification performance was fair for the equidose (10 Gy) (AUC = 0.73).

Statistically significant spectral features were identified and compared to authentic standards to match *m*/*z*, retention time, and tandem MS/MS spectra for unambiguous identification (Table 1 and Table S1, Figure 2 and Figure S3). Urinary carnitine concentration increased at 4 h in WT mice but showed no change in p53^−/−^ mice. Other compounds showed similar patterns between WT and p53^−/−^ mice; however, these were only statistically significant in WT mice (except N1-acetylspermidine at 24 h in p53^−/−^) (Figure 2 and Figure S3). The greatest change was observed for creatine, which decreased at 4 h but increased nearly 5-fold at 24 h for WT. Similarly, phenylacetylglycine and 4-pyridoxic acid decreased slightly at 4 h and increased at 24 h. 1-Methylhistamine, 1-methylnicotinamide, and N1-acetylspermidine showed similar patterns, where all increased at 24 h for both WT and p53^−/−^ groups, with slightly higher fold changes observed in WT. Minor decreases were observed at 24 h for hippuric acid. Kynurenic acid and 3-methoxy-4-hydroxyphenylethyleneglycol sulfate (MHPG-SO_4_) slightly decreased at 4 h. Pathway enrichment showed primary perturbations to fatty acid β oxidation, tryptophan, vitamin B6, and histamine pathways, amino acid metabolism, glycine conjugation, and redox metabolism (Figure 3, Table 1). Perturbation in carnitine synthesis and fatty acid oxidation was only observed in WT (Figure 2). ROC curve analysis showed the highest specificity and sensitivity between WT and p53^−/−^ mice at 4 h for carnitine and N1-acetylspermidine combined (AUC = 0.91) at the equitoxic dose (8 Gy) and fair values for the equidose (10 Gy, AUC = 0.73) (Figure 4). At 24 h, classification performance did not increase through combining metabolites. AUC values were fair for carnitine (AUC = 0.75) and N1-acetylspermidine (AUC = 0.79) at the equitoxic dose (8 Gy) but genotypes were not differentiated by creatine (AUC < 0.50). Better specificity and sensitivity were observed for creatine (AUC = 0.84) and N1-acetylspermidine (AUC = 0.81) for the equidose (10 Gy), but these were poor for carnitine (AUC = 0.62) (Figure S4).

## 4. Discussion

In the present study, we compared changes in the urinary metabolome at 4 and 24 h post-irradiation in WT (8 and 10 Gy) and p53^−/−^ (10 Gy) mice with IR induced hematopoietic injury. Similarities in metabolic responses after IR exposure between genotypes suggest presence of in vivo compensatory mechanisms and highlight the utility of genetically engineered mouse models in biodosimetry studies compared to in vitro models [30,31]. After IR exposure, an overall “muted” response was observed in p53^−/−^ genotype compared to WT, likely due to decreased hematopoietic stem cell apoptosis [15]. Perturbation in carnitine was only observed in WT mice, while similar perturbations were observed in metabolites involved in tryptophan, vitamin B6, and histamine pathways, glycine conjugation, and redox metabolism between genotypes. These results suggest these pathways are not downstream of p53 regulation (e.g., dietary or microbial origin) or are balanced through compensatory mechanisms. While similar metabolite patterns were observed, post-irradiated genotypes were differentiated by ROC curve analysis with high to fair specificity and sensitivity for carnitine, N1-acetylspermidine, and creatine. Genotype-independent urinary markers may represent targets for universal biodosimetry. However, these results show some markers are genotype-dependent and must be addressed in biodosimetry models.

While genetically engineered knockout mouse models are an extreme example of genetic variability in the general population, they provide vital information on possible effects to biodosimetry accuracy. Considering the current study, IR exposure will quickly elicit p53-mediated apoptosis in sensitive WT tissues along with a cascade of downstream gene activation in other tissues (see review [32]). Mutations in p53 can dampen these effects and lead to deficient tumor suppression through loss (or gain) of function due to missense mutations caused by environmental mutagens or dominant negative effects in heterozygous (e.g., LFS) individuals. While lowered apoptotic response may confer hematopoietic radioresistance after a potential radiological event, here observed as reduced metabolite fold change, inadequate DNA repair and cell death may lead to downstream complications such as cancer development. Radiosensitive mouse models also show differential transcriptomic and metabolomic responses after IR exposure. A similar yet “heightened” change in gene expression in Parp1^−/−^ was observed compared to WT mice [33]. Conversely, overall gene repression was observed in Atm^−/−^ mice, including the resultant p53 response (e.g., p53-regulated genes Cdkn1a, Aen, Phlda3, Bbc3, and Mdm2) [34]. The urinary small molecule signatures in both these models indicated differential phenotypic perturbation compared to WT, primarily as higher fold changes across validated metabolites (Atm^−/−^ mice) and in TCA cycle intermediates (Parp1^−/−^ mice) [10,12].

Some common metabolites identified between genetic mutant mouse studies include hippuric acid (Atm^−/−^), kynurenic acid (Atm^−/−^), and 4-pyridoxic acid (Parp1^−/−^). Decreased urinary levels of kynurenic acid have been consistently reported in murine models after IR exposure [35], as has perturbation to several other intermediaries of tryptophan metabolism (e.g., xanthurenic acid). Approximately 95% of free tryptophan is metabolized via the kynurenine pathway, and as such, has become a common target for several diseases [36]. 4-Pyridoxic acid is metabolized from vitamin B6 in the liver by aldehyde oxidase for elimination in urine, but may also be a microbial product by action of pyridoxal 4-dehydrogenase. Interestingly, the conversion of histidine to histamine requires the active metabolite of vitamin B6 (pyridoxal-5-phosphate) and both 4-pyridoxic acid and 1-methylhistamine levels increase at 24 h in the current study [37]. As a marker of vitamin B6 catabolism, 4-pyridoxic acid has been implicated in several disease processes as well, from cardiovascular to bowel disease [38]. Hippuric acid is formed by microbial conjugation of glycine and benzoic acid and may promote glycine homeostasis. A common issue that may arise with the utility of these compounds in biodosimetry is variation due to their dietary or host microbial origin [39]; however, the role of the host microbiota during ARS is an understudied field deserving of more attention [40].

Three additional compounds (phenylacetylglycine, 1-methylhistamine, and 1-methylnicotinamide) have been previously identified in radiation exposure studies and show similar fold changes between p53^−/−^ and WT mice. Phenylacetylglycine is a conjugate of glycine and phenylacetic acid formed by glycine N-acyltransferase and similarly to hippuric acid plays a role in detoxification through the homeostasis of intracellular benzoic acid and glycine levels. The role radiation plays in glycine conjugate excretion is an interesting topic, especially when considering its implications in free coenzyme A levels and downstream metabolism [41]. Histamine methyltransferase forms 1-methylhistamine from histamine and was identified in B6C3F1/Crlj mice after 4 Gy IR exposure [42]. Histamine and inflammation in ARS have long been established [43] and histamine has been proposed as an agent to increase radiosensitivity and improve radiotherapy [44]. The possible role of histamine in suppressing apoptosis after IR exposure may explain the lack of a statistically significant response in p53^−/−^ mice at 24 h compared to WT mice [45]. Disruption to nicotinic acid and nicotinamide metabolism has also been observed in radiotherapy [46] and may affect downstream redox metabolism and NAD^+^ levels. Nicotinamide is a product of sirtuin (SIRT) deacetylation and converted to 1-methylnicotinamide through nicotinamide N-methyltransferase (NNMT). While p53 is a well described target of SIRT1 deacetylation [47], the ubiquity of proteins acquiring these modifications may contribute to the similar fold change observed in 1-methylnicotinamide. One novel metabolite of IR exposure, MHPG-SO_4_, was detected in the current study. MHPG-SO_4_ is a major metabolite of norepinephrine, which is a major catecholamine neurotransmitter along with dopamine and epinephrine. Increased catecholamine excretion has been observed in humans after accidental irradiation [48] but little information exists on their metabolites after IR exposure.

While similar patterns emerge for metabolite perturbation between genotypes, classification models show how false negatives in the p53^−/−^ genotype may complicate prediction of IR toxicity. Three metabolites (carnitine, N1-acetylspermidine, and creatine) identified in the current study are involved downstream of p53-targeted pathways (see below) and show high to fair classification performance across time points. Elevated carnitine levels may indicate fatty acid β oxidation perturbation, as carnitine palmitoyltransferase 1 (CPT1) binds carnitine to a corresponding fatty acid to form an acylcarnitine for transportation across mitochondrial cell membranes. Deficiency of p53 may affect both substrate concentration and enzymatic conversion, as it upregulates CPT1C, a brain specific isoform, by phosphorylating activated protein kinase (AMPK) [49] and also represses sterol regulatory element-binding protein 1 (SREBP-1) and downstream fatty acid synthesis [50] (see review [51]). Increased murine urinary N1-acetylspermidine after IR exposure is independent of age and previous exposure [52] and has been well described [53]. Spermidine N-acetylation is catalyzed by spermidine/spermine N1-acetyltransferase 1 (SAT1), a gene induced by p53 and participates in ferroptotic cell death [54]. Similarly, urinary levels of creatine are commonly found increased after IR exposure, likely due to a combination of malnutrition, muscle wasting, and kidney dysfunction [55]. Muscle creatine kinase (MCK) is one of the earlier p53-target genes identified [56], but p53 deficiency in skeletal muscle mass and function is unclear [57,58]. Here, while lower concentrations of these three metabolites in the p53^−/−^ genotype may distinguish from WT mice, similar patterns for N1-acetylspermidine and creatine do suggest compensatory pathways in p53-deficient mice. For an example considering N1-acetylspermidine, acetylated and non-acetylated spermine and spermidine steady-state levels were found to remain unchanged in radiosensitive shSAT cells, possibly through altering ornithine decarboxylase activity [59]. In terms of IR exposure, these results are encouraging for developing universal biodosimetry marker panels for urine but reduced model accuracy due to genetic variability must be recognized.

## 5. Conclusions

Differences in tissue and cell response to IR exposure in p53-deficient mice will result in decreased mortality due to hematopoietic injury compared to WT. Conversely, at higher doses (>15 Gy) increased mortality from GI injury occurs due to lack of cell arrest and mitotic catastrophe in crypt cells. Identification of IR induced hematopoietic injury biomarkers is extremely relevant in a radiological emergency, as significant reduction in mortality may be achieved with proper supportive care. This study, along with previous examples of radiosensitive genotypes (Parp1^−/−^ and Atm^−/−^), demonstrates how genetic variability in the general population may affect predictive performance in small molecule-based biodosimetry. As there is likely no single metabolic biomarker indicative of IR exposure and ARS severity, the goal is to combine small molecules with other targets (e.g., proteins or microRNA expression) to increase the predictive performance of biodosimetry models for complex exposures. However, both attenuated and dampened metabolite responses pose challenges with false positive or negative rates, respectively. These challenges may be further exacerbated when moving from rather simple exposures (whole-body photon irradiation) to more complex exposures (partial body shielding and mixed neutron/photon components). Future work will continue to assess genotypic independence in complex exposure and further biodosimetry marker panel development.

## Figures and Tables

**Table 1:** Validated urinary metabolites at 4 and 24 h in wild-type and p53^−/−^ mice after ionizing radiation exposure.

Metabolite	Adduct	RT (min)	Experimental (*m*/*z*)	Calculated (*m*/*z*)	Mass Error (ppm)	Formula	HMDB ID	Metabolic Pathway
Carnitine	[M + H]^+^	0.29	162.1137	162.1130	4.3	C_7_H_15_NO_3_	0000062	Fatty acid β oxidation
Kynurenic acid	[M + H]^+^	1.36	190.0512	190.0504	4.2	C_10_H_7_NO_3_	0000715	Tryptophan metabolism
Hippuric acid	[M + H]^+^	1.75	180.0669	180.0661	4.4	C_9_H_9_NO_3_	0000714	Phenylalanine metabolism/Glycine conjugation
Phenylacetylglycine	[M + H]^+^	2.33	194.0817	194.0817	0.0	C_10_H_11_NO_3_	0000821	Phenylalanine metabolism/Glycine conjugation
Creatine	[M + H]^+^	0.29	132.0780	132.0773	5.3	C_4_H_9_N_3_O_2_	0000064	Amino acid metabolism
4-Pyridoxic acid	[M + H]^+^	0.34	184.0603	184.0610	3.8	C_8_H_9_NO_4_	0000017	Vitamin B6 metabolism
MHPG-SO_4_	[M − H]^−^	0.39	263.0224	263.0226	0.6	C_9_H_12_O_7_S	0000559	Noradrenaline metabolism
1-Methylhistamine	[M + H]^+^	0.25	126.1033	126.1031	1.4	C_6_H_11_N_3_	0000898	Histidine metabolism
1-Methylnicotinamide	[M + H]^+^	0.28	137.0722	137.0715	5.2	C_7_H_8_N_2_O	0000699	Nicotinate and nicotinamide metabolism
N1-Acetylspermidine	[M + H]^+^	0.25	188.1769	188.1763	3.3	C_9_H_21_N_3_O	0001276	Polyamine metabolism

* Metabolic pathways identified in MetaboAnalyst using the Kyoto Encyclopedia of Genes and Genomes (KEGG), The Small Molecule Pathway Database, and the Human Metabolome Database (HMDB).

## Supplementary Materials

The following are available online at https://www.mdpi.com/2218-1989/10/6/234/s1. Table S1: *p*-values for validated metabolites. Figure S1: Study design and sample size. Figure S2: PCA plot of unirradiated genotypes (WT, p53^+/−^, and p53^−/−^). Figure S3: Heatmap of urinary metabolites in wild-type (WT) mice (8 Gy) and p53^−/−^ mice (10 Gy) at 4 and 24 h post-irradiation. Figure S4: ROC curves for select metabolites at 24 h.
