# Supplementary material for: Effects of Genetic Variation on Urinary Small Molecule Signatures of Mice after Exposure to Ionizing Radiation: A Study of p53 Deficiency
Source: Metabolites. 2020 Jun 8;10(6):234. doi: 10.3390/metabo10060234 (PMC7345090; doi:10.3390/metabo10060234)
Supplement: Supplementary file 1 [file metabolites-10-00234-s001.pdf]

Supplementary Table 1. False-discovery rate corrected p-values determined from Welch's t-test.

Metabolite	p-value WT 4 h 8 Gy	p-value WT 24 h 8 Gy	p-value WT 4 h 10 Gy	p-value WT 24 h 10 Gy	p-value KO 4 h 10 Gy	p-value KO 24 h 10 Gy
Carnitine	**0.015**	0.404	0.076	0.645	0.528	0.295
Kynurenic acid	**0.004**	0.274	0.152	0.384	0.387	0.687
Hippuric acid	0.188	0.051	0.860	**0.039**	0.854	0.272
Phenylacetylglycine	**0.006**	0.712	0.939	**0.025**	0.314	0.356
Creatine	**0.006**	**0.011**	0.411	**0.001**	0.275	0.097
4-Pyridoxic acid	**0.042**	0.061	0.300	**0.025**	0.275	0.108
MHPG-SO_4	**0.003**	0.205	**0.005**	0.572	0.223	0.224
1-Methylhistamine	0.952	**0.046**	0.571	**0.002**	0.532	0.267
N-Methylnicotinamide	0.920	**0.016**	0.959	**0.005**	0.818	0.128
N1-Acetylspermidine	0.826	**0.007**	0.876	**0.001**	0.349	**0.014**

SUPPLEMENTARY FIGURE 1

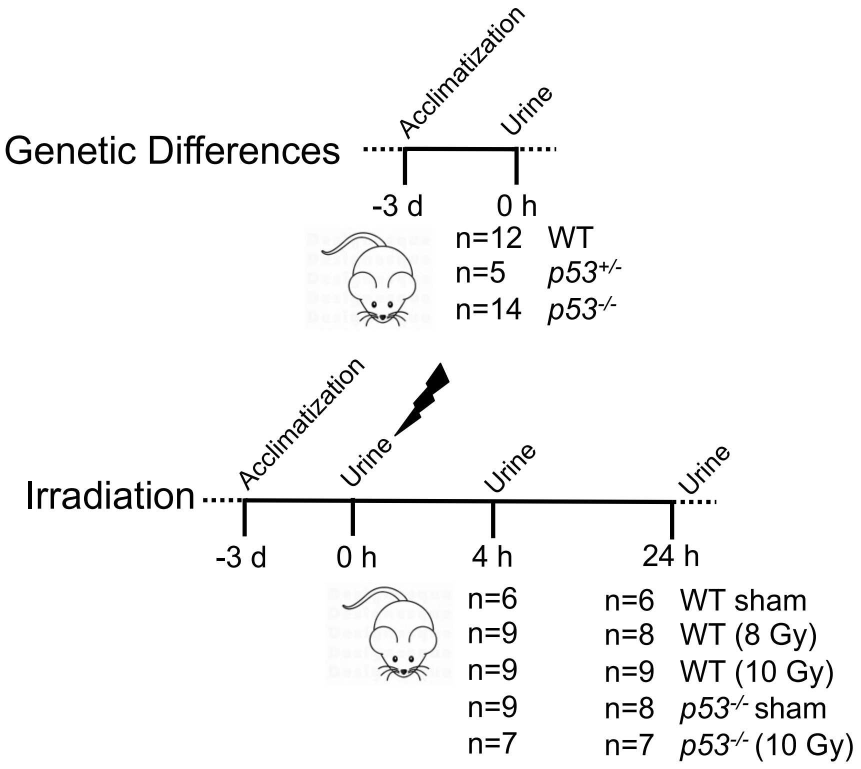

Supplementary Figure 1. Study design and sample size. The 4 and 24 h sham urine were combined for wild-type and *p53*$^{-/-}$ for their respective control group.

SUPPLEMENTARY FIGURE 2

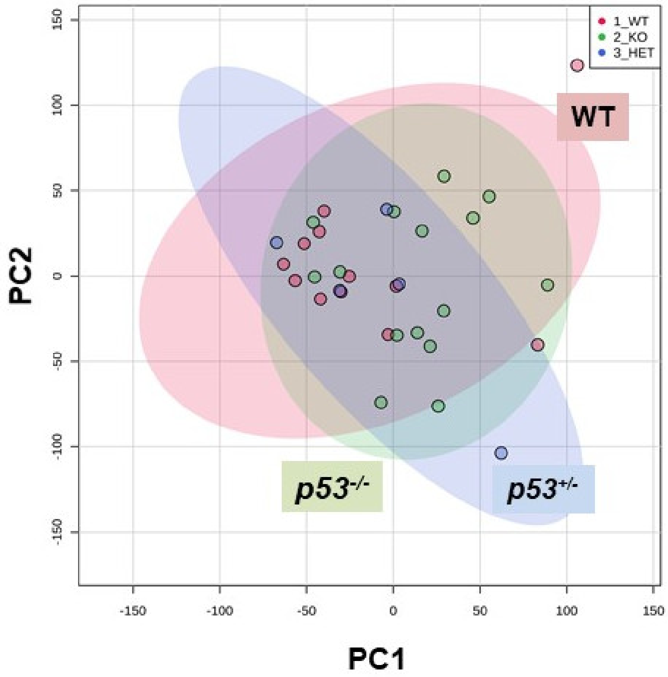

Supplementary Figure 2. Principal component analysis of unirradiated genotypes (WT, *p53*$^{+/-}$, and *p53*$^{-/-}$ mice) showing high overlap, thus minimal differences, between groups.

SUPPLEMENTARY FIGURE 3

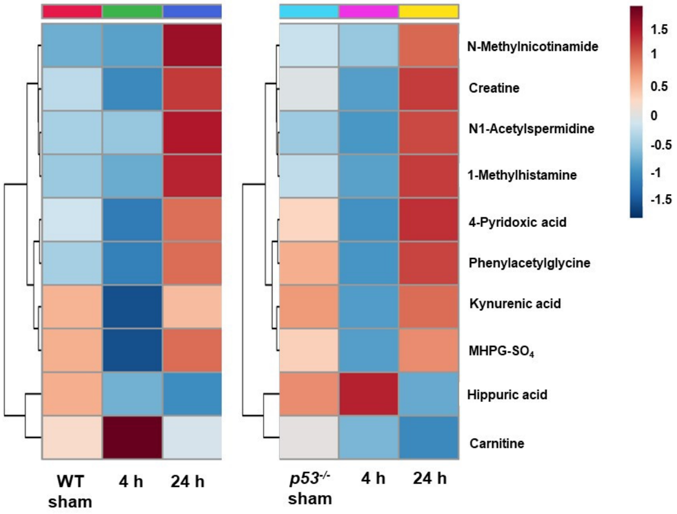

Supplementary Figure 3. Heatmap of urinary metabolites in wild-type (WT) mice (8 Gy) and *p53*$^{-/-}$ mice (10 Gy) at 4 and 24 h post-irradiation.

SUPPLEMENTARY FIGURE 4

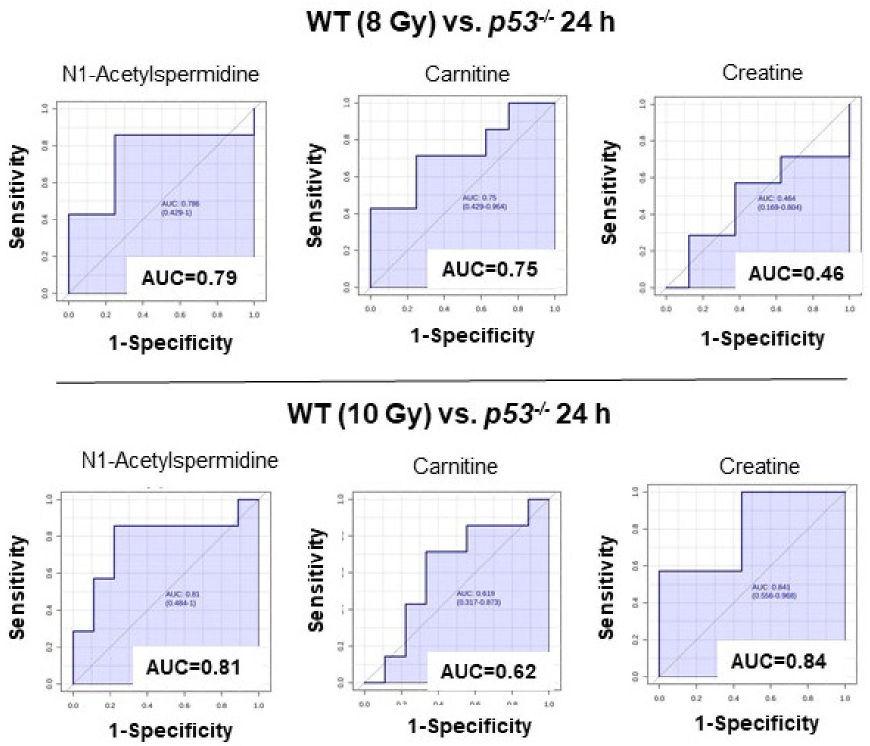

Supplementary Figure 4. Specificity and sensitivity of N1-acetylspermidine, carnitine, and creatine at 24 h post-irradiation determined by the area under the curve (AUC) values from receiver operating characteristic (ROC) analysis. The equidose (10 Gy) shows better classification performance than the equitoxic dose (8 Gy); however, poor specificity and sensitivity was observed (AUC<0.60) when metabolites were combined.
